# Healthy dietary patterns and ovarian cancer risk and survival: a systematic review and meta-analysis

**DOI:** 10.3389/fnut.2025.1681162

**Published:** 2025-10-13

**Authors:** Yiyi Xu, Jiner Chen, Ke Zhao

**Affiliations:** Department of Obstetrics and Gynecology, The Sixth People’s Hospital of Zhuji, Zhuji, Zhejiang, China

**Keywords:** dietary patterns, ovarian cancer, survival, systematic review, meta-analysis

## Abstract

**Background:**

Studies investigating the associations between healthy dietary pattern and risk and survival of ovarian cancer have been limited and inconsistent. Therefore, we carried out this comprehensive systematic review and meta-analysis to analyze the available literature on the associations between healthy dietary patterns and risk and survival of ovarian cancer.

**Methods:**

PubMed, Web of Science, Scopus and China National Knowledge Infrastructure (CNKI) were comprehensively searched for the relevant articles published from databases inception to October 2024. According to heterogeneity, the pooled relative risks (RRs) and 95% confidence intervals (CIs) were calculated for the highest versus the lowest categories of healthy dietary patterns in relation to ovarian cancer risk and survival, using the random-effects or fixed-effects meta-analyses.

**Results:**

Sixteen studies (12 cohort and four case-control studies) with 615,203 participants, 5,452 ovarian cancer cases and 3,028 ovarian cancer deaths were included in the final analysis. Combining 15 effect sizes from eight studies, we found the evidence of a reduced risk of ovarian cancer in the highest compared with the lowest categories of healthy dietary patterns (RR = 0.91; 95%CI: 0.85–0.98, *P* = 0.013). The pooled analyses also revealed that healthy dietary patterns was associated with improved ovarian cancer survival (RR = 0.85; 95% CI:0.0.76–0.95, *P* = 0.004), with significant heterogeneity (I^2^ = 54.3%, *P* = 0.004). Moreover, per SD increment in healthy dietary score was related to a 14% reduced risk of ovarian cancer mortality (RR = 0.86; 95% CI: 0.81–0.91, *P* < 0.001).

**Conclusion:**

Our findings demonstrated that high adherence to the healthy dietary patterns was associated with a reduced risk and improved survival of ovarian cancer. Future large-scale prospective studies are required to confirm and strengthen these findings.

## Introduction

According to the GLOBOCAN cancer statistics in 2020, ovarian cancer is the second leading cause of mortality among gynecologic malignancies worldwide, with an estimated 313,959 new cases and 207,252 deaths (1). In the United States, the latest data shows that ovarian cancer is the leading cause of gynecological cancer-related deaths, with 19,680 new cases and 12,740 deaths in 2024 (2). By comparison, the incidence rate of ovarian cancer in China is relatively low, with an age-standardized rate of 5.32 per 100,000 (3). Despite significant improvements in current diagnostic techniques and treatments, more than 75% of patients with ovarian cancer are initially diagnosed when it has advanced and they have a 5-year relative survival rate of 29% (4). The well-known risk factors of ovarian cancer included absence of pregnancy, early age of menarche, late age at menopause, use of estrogen and hormone-replacement therapy, and family history of ovarian cancer (5). Therefore, the identification of modifiable risk factors is especially importance for the primary prevention of ovarian cancer.

During the past few decades, increasing substantial evidence shows that dietary factors play a critical part in the etiology of ovarian cancer (6). Previous epidemiological studies have predominantly examined the associations between individual nutrients, foods and risk and survival of ovarian cancer (7–10). For example, evidence show that high consumption of vegetables, but not of fruits, are associated with a reduced risk of ovarian cancer (7). However, in reality, people do not eat nutrients or foods alone, but consume meals containing combinations of many nutrients and foods that possibly interact with each other (11). Considering the complexity of individual’s diet and potential interactions between food components, dietary pattern analysis has emerged in nutritional research as a more holistic research approach to evaluate the relationship between overall diet and various chronic non-communicable diseases, including ovarian cancer (12).

Healthy dietary patterns, which are generally characterized by high consumption of fruits, vegetables, fish, whole grains and low consumption of red meat, processed meat and refined grains, have been recommended for cancer prevention and survivorship (13, 14). Nevertheless, little is known about the impact of dietary patterns on ovarian cancer survival. Over the past decade, extensive attention has been focused on the role of dietary patterns specifically in ovarian cancer survival (15). Up to day, numerous epidemiological studies have shown the associations between overall dietary patterns and risk and survival of ovarian cancer (15–22), but their conclusions are still inconsistent. Even though some studies have shown the significant protective role of adherence to the healthy dietary patterns against ovarian cancer (18, 23), other studies found the apparent positive or null findings (16, 17, 21, 22). In addition, the continuous update project (CUP) by the World Cancer Research Fund/American Institute for Cancer Research (WCRF/AICR) did not make the firm conclusions about the links between dietary patterns and ovarian cancer (24). Notably, a previous meta-analysis of three observational studies showed no significant association between healthy dietary pattern and ovarian cancer risk (25). Alizadeh et al.’ meta-analysis mainly focused on ovarian cancer risk and only included three studies (1 cohort and 2 case-control studies). Furthermore, to the best of our knowledge, no meta-analysis thus far has been conducted to comprehensively assess the associations between healthy dietary patterns and survival of ovarian cancer. To fill in this literature gap, we carried out a systematic review and meta-analysis was to comprehensively review and synthesize the up-to-date evidence from previous studies published up to October, 2024 and to further ascertain the exact associations between healthy dietary patterns and risk and survival of ovarian cancer using meta-analysis.

## Methods

### Literature search strategy

This systematic review was performed in accordance with the Preferred Reporting Items for Systematic Reviews and Meta-Analysis (PRISMA) guidelines (26). The study protocol was registered in the International Prospective Register of Systematic reviews (PROSPERO; registration number CRD42016036157). A comprehensive literature search was conducted across PubMed, Web of Science, Scopus and China National Knowledge Infrastructure (CNKI) databases for articles published up to 31 October 2024, without restrictions on language and publication data. The following search terms were used: (diet OR dietary score OR diet indices OR dietary quality OR dietary index OR dietary pattern OR eating pattern OR food pattern) AND (ovarian neoplasm OR ovarian cancer OR ovarian carcinoma OR ovarian tumor OR ovarian mass OR ovary neoplasm OR ovary cancer OR ovary carcinoma). Reference lists of retrieved articles, reviews and meta-analyses were manually screened to identify additional relevant studies. Unpublished studies or grey literature were not eligible in this study. The complete search strategy is detailed in Supplementary Table 1.

### Studies inclusion criteria

Two authors (K.Z. and J.-E.C) independently performed the literature search and reviewed the titles and abstracts of retrieved articles reporting the relationship between healthy dietary patterns and risk and survival of ovarian cancer. Any disagreements between two authors were solved by consultation with the corresponding author (Y.-Y.X). When all authors agreed, the full-text versions of published articles were reviewed against inclusion and exclusion criteria for this meta-analysis. Studies were eligible for inclusion if they met the following criteria: (1) observational studies, e.g., case-control and cohort studies, conducted in adult population (aged ≥ 18 years); (2) the exposure of interest was healthy dietary patterns; (3) the main outcome of interest was ovarian cancer risk and survival; (4) providing risk estimates [odds ratios (ORs), relative risks (RRs), hazard ratios (HRs)] and their corresponding 95% confidence intervals (CIs) (or sufficient data to calculate them) for the relationship between healthy dietary patterns and ovarian cancer risk or survival; (5) if retrieved article lacked sufficient detail, we would contact the corresponding author of eligible studies by email. In addition, studies were excluded if they met one of the following criteria: (1) irrelevant articles; (2) non-observational studies, such as reviews, editorials, case reports and conference letters; (3) lack of sufficient data to gain HRs, RRs or ORs with 95% CIs. The PECOS criteria for inclusion and exclusion of studies is summarized in Table 1.

**TABLE 1:** Inclusion and exclusion of studies using the PECOS criteria.

Population	Adults 11
Exposure	Healthy dietary patterns (index-based or data-driven)
Comparator	Highest vs. lowest categories of exposure
Outcomes	Ovarian cancer risk and survival
Study design	Cohort, case-control or cross-sectional studies

PECOS, population, exposure, comparator, outcome, and study design.

### Data extraction

Two authors (K.Z and J.-E.C) independently gathered the following information: first author’s last name, publication year, country, study design, total number of participants, numbers of ovarian cancer cases and/or deaths, mean age/age range, method of dietary assessment, confounding factors that were most-adjusted in the multivariate analyses, identification of healthy dietary patterns and effect sizes (RRs, HRs or ORs and their corresponding 95%CIs). In the case of presenting pre- and post-diagnosis stratified effect sizes, we treated them as two separate studies in the final analysis. Discrepancies in data extraction between the authors were resolved by consensus or discussion with the corresponding author (Y.-Y.X).

### Quality assessment

The Newcastle-Ottawa Scale (NOS) was employed to assess the quality of included non-randomized studies in previous meta-analyses (27). In the NOS checklist, scores ranged from 0 to 9 based on the eight items related to three dimensions: study selection (4 stars), comparability of participants (2 stars), and assessment of outcome/exposure of interest (3 stars). Finally, studies with NOS scores ≥ 7 were deemed to be of high methodological quality (28).

### Assessment of heterogeneity

Heterogeneity across studies was explored with the use of the Cochran’s Q test and *I*^2^ statistic. A *p*-value for heterogeneity < 0.10 and I^2^ > 50% were considered to show significant heterogeneity among the included studies, in which case a random-effects model was used to calculate the pooled RRs. Otherwise, the fixed-effects model was used (29).

### Statistical analysis

Given the low prevalence of ovarian cancer in humans, ORs in case-control studies were directly considered equivalent to RRs (30). We pooled the RRs from eligible studies using random-effect or fixed-effect models. If the results showed significant heterogeneity across studies, sensitivity and subgroup analyses were used to determine the possible reasons contributing to heterogeneity. In our analyses, subgroup analyses were stratified by study region (Asian and Western countries), methods used to determine healthy dietary patterns (*a priori* and *a posteriori*), follow-up time (< 10 and ≥ 10 years), mean age (< 50 and ≥ 50 years), and sample size (< 5,000 and ≥ 5,000). Sensitivity analysis was undertaken to explore the influence of each study on the pooled risk estimates by sequential exclusion of each study at a time. If at least 10 studies were available, potential publication bias was tested by visual inspection of funnel plots and formal testing for “funnel plot” asymmetry using Begg’s and Egger’s tests (31). When publication bias was detected, the trim and fill method was used to correct the results (32). All statistical analyses were performed using STATA 18.0 (StataCorp, College Station, Texas, United States). A two-sided *P-*value of < 0.05 was considered statistically significant except where otherwise specified.

## 3 Results

### 3.1 Overview of included studies for the systematic review and meta-analysis

**FIGURE 1:**
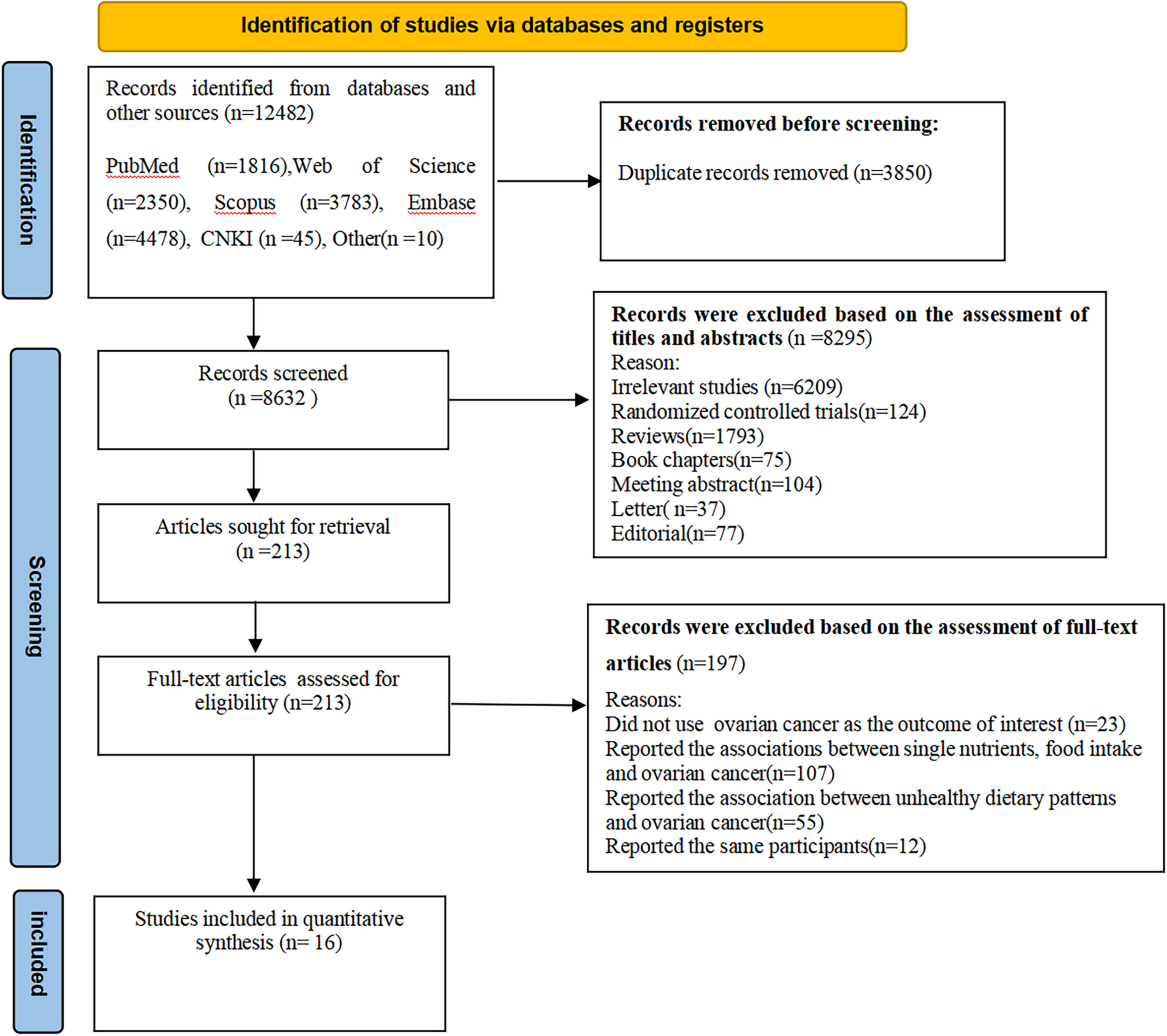
Flow chart of article screening and selection process.

A flowchart of the study selection process is shown in Figure 1. In total, we identified 12,482 articles through five databases search and reference lists of retrieved articles, published reviews and meta-analyses. Subsequent to the removal of 3,850 duplicates, 8,632 articles remained for further screening the titles and abstracts. After evaluating the titles and abstracts, 8,295 articles were excluded. Of the remaining 213 full-text articles, 197 articles were excluded because of the following reasons: the outcome of interest was not ovarian cancer (*n* = 23), reported the associations between single nutrients, food intake and ovarian cancer (*n* = 107), reported the association between unhealthy dietary patterns and ovarian cancer (*n* = 55), and reported the same participants (*n* = 12). Finally, sixteen articles, including 12 prospective cohort (15, 17, 19, 20, 22, 23, 33–35, 36–38) and 4 case-control studies (16, 18, 21, 39) were included in the final analysis.

### Study characteristics

The characteristics of all included studies are shown in Table 2. Sixteen articles with 615,203 participants, 5,452 ovarian cancer cases and 3,028 ovarian cancer deaths were included in this systematic review and meta-analysis. The majority of the included studies were prospective cohort studies (15, 17, 19, 20, 22, 23, 33–35, 36–38), and four studies were case-control studies (16, 18, 21, 39). Publication date of all included studies ranged from 2008 to 2024. The age of participants ranged from ages 18 to above. The number of participants in included studies ranged from 483 to 161,816, and the follow-up duration for cohort studies from 3.7 to 24 years. Of the 16 included studies, 10 studies were conducted in the United States (15, 17, 19–22, 34, 35, 38, 39), two in China (36, 37), two in Australia (16, 33), one in Italy (18), and one in Canada (23). All of included studies used food questionnaire questionnaires (FFQs) to collect dietary intake data (15–23, 33–39). According to NOS criteria, all the included studies were classified as of high quality (15–23, 33–39). The quality assessment of included studies bases on NOS criteria is shown in Table 3.

**TABLE 2:** Characteristics of included studies on the associations between healthy dietary patterns and ovarian cancer risk and survival.

References	Country	Study design	Total number of participants	Mean age/age range	Dietary assessment	Adjustment or matched for in analyses	Effect sizes OR/RR (95% CI)
Sasamoto et al. (15)	United States	Cohort	1,003 (695 deaths)	17–79 years	FFQ	Age at diagnosis, calendar year at diagnosis, histology, stage, smoking status, body mass index, total energy intake, non-steroidal anti-inflammatory drug (NSAID) use.	Pre-diagnosis AHE HR: 0.95 (0.73–1.22); Post-diagnosis AHEI HR: 1.12 (0.79–1.60)
Kolahdooz et al. (16)	Australia	Case-control	1,460 (683 cases)	18–79 years	FFQ	Age (in y), plus oral contraceptive use (none, < 60, or ≥ 60 months), parity (0, 1–2, or 3), education after high school (yes or no), and energy intake (log transformed).	Fruit and vegetable: OR: 0.95 (0.69–1.31)
Chang et al. (17)	United States	Cohort	97,292 (311 cases)	≥ 20 years	FFQ	Race/ethnicity, total energy intake, parity, oral contraceptive use, lifetime strenuous physical activity, menopausal status/hormone therapy use, and wine intake; with age as time-scale and stratified by age at baseline.	Plant-based: RR: 1.65 (1.06–2.54)
Edefonti et al. (18)	Italy	Case-control	4,444 (1031 cases)	17–79 years	FFQ	Age, education, parity, menopausal status, geographic area, body mass index, history of female cancers, history of digestive cancers, energy intake.	Vitamins and fiber: OR: 0.77 (0.61–0.98)
Armidie et al. (19)	United States	Cohort	483 (310 deaths)	20–79 years	FFQ	Age (years), education, annual household income, physical activity in the year before diagnosis, smoking status (never, current, or former smoker), study site (Southwest, Southeast, or North), and histotype (HGSOC or other).	AHEI-2010 HR: 0.89 (0.83–1.16), AHEI-2020 HR:0.78 (0.56–1.08)
Wen et al. (20)	United States	Cohort	853 (130 deaths)	18–79 years	FFQ	Age at diagnosis, total energy intake, body mass index, diet change, comorbidities, education, FIGO stage, histological type, histopathologic grade, menopausal status, parity, oral contraceptives, physical activity, residual lesions, smoke status, other dietary patterns.	Pre-diagnosis healthy pattern HR:0.54 (0.30–0.98)
Qin et al. (21)	United States	Case-control	1,044 (415 cases)	22–79 years	FFQ	Age, region, education, parity, oral contraceptive use, menopause status, tubal ligation status, first-degree family history of breast/ovarian cancer, body mass index, physical activity, and total energy intake.	AHEI-2010 OR:0.66 (0.45–0.98); HEI-2005 OR:0.83 (0.56–1.23); HEI-2010 OR:0.74 (0.50–1.11).
Xie et al. (22)	United States	Cohort	82,948 (696 cases)	30–55 years	FFQ	Age (months), total energy intake (kcal/d), family history of ovarian cancer (yes, no), tubal ligation, BMI (kg/m^2^), parity (yes, no), number of additional pregnancies (continuous), oral contraceptive use duration, smoking, menopausal status, type and duration of PMH use, age at menarche (years), hysterectomy, unilateral oophorectomy, lactose intake (g/d), caffeine intake (mg/d), and physical activity.	AHEI-2010 HR:1.03 (0.84–1.34); HEI-2005 HR:0.85 (0.65–1.12); aMED: HR:0.91 (0.71–1.18).
Arthur et al. (23)	Canada	Cohort	2,735 (100 cases)	44–70 years	FFQ	Age at entry and adjusted for education, non-alcohol energy intake, smoking status, alcohol intake, BMI, diet score, physical activity, age at menarche, parity, menopause, HRT use, oral contraceptive use.	HLI HR:0.50 (0.27–0.92)
Al Ramadhani et al. (33)	Australia	Cohort	650 (278 deaths)	20–79 years	FFQ	Age (continuous), log energy (continuous), smoking status at 12 months (never/former/current), and FIGO stage, and stratified by physical activity at 12 months.	Pre-diagnosis HEI-2010 HR1.08 (0.80–1.48), AHEI-2010 HR:1.12 (0.84–1.51); Post-diagnosis HEI-2010 HR:1.33 (0.89–2.01), AHEI-2010 HR:1.22 (0.80–1.84)
Arthur et al. (34)	United States	Cohort	108,136 (904 cases)	50–79 years	FFQ	Age at entry, education, non-alcohol energy intake, ethnicity, age at menarche, parity, combined estrogen and progesterone therapy, unopposed estrogen therapy, oral contraceptive use, family history of ovarian cancer, and age at menopause.	HLI HR:0.96 (0.77–1.19)
Cao et al. (35)	United States	Cohort	150643 (1,107 cases, 893 deaths)	50–71 years	FFQ	Baseline age, race/ethnicity, residency, education level, marriage status, number of liveborn, age at menarche, post-menopausal, family history of any cancer, HRT usage, oral contraceptives, comorbidities, leisure-time physical activity, smoking, BMI, and total energy intake (kcal/day).	Morbidity HEI-2015 HR: 1.03 (0.84–1.26); aMED HR:1.03 (0.84–1.27); DASH: HR:0.83 (0.68–1.02); Mortality HEI-2015 HR:0.75 (0.59–0.95); aMED HR:0.68 (0.53–0.87); DASH: HR:1.01 (0.80–1.29).
Chandran et al. (39)	United States	Case-control	595 (205 cases)	20–79 years	FFQ	Age, education, race, age at menarche, menopausal status, parity, oral contraceptive use (ever, never), HRT use (never, unopposed estrogen only, any combined HRT), tubal ligation (no, yes), BMI (continuous), total calories (continuous), physical activity, smoking status, and pack years smoked (continuous).	HEI-2005 OR:0.90 (0.55–1.74)
Chen et al. (36)	China	Cohort	560 (211 deaths)	18–79 years	FFQ	Age at diagnosis, pre/post-diagnosis body mass index, pre/post-diagnosis total energy intake, pre/post-diagnosis cigarette smoking, education, income, pre/post-diagnosis physical activity, menopausal status, histological type, FIGO stage, comorbidities, and residual lesions.	Pre-diagnosis AMED HR: 0.59 (0.38–0.90); Post-diagnosis AMED HR:0.61 (0.41–0.91)
Liu et al. (37)	China	Cohort	549 (206 deaths)	18–79 years	FFQ	Age at diagnosis, pre/post-diagnosis body mass index, pre/post-diagnosis total energy intake, pre/post-diagnosis physical activity, pre/post-diagnosis smoking status, education, income, FIGO stage, histological type, and residual lesions.	Pre-diagnosis HEI-2020 HR: 0.66 (0.46–0.93); Post-diagnosis HEI-2020 HR:0.68 (0.49–0.96)
Thomson et al. (38)	United States	Cohort	161,808 (305 deaths)	50–79 years	FFQ	Age at diagnosis (continuous), stage at diagnosis (localized, regional, distant), race/ethnicity, diabetes, physical activity, total energy intake (quintiles), waist circumference, family history of ovarian cancer, and clinical trial arms	HEI HR:0.75 (0.55–1.01)

BMI, body mass index; DII, dietary inflammatory index; FFQ, food frequency questionnaire; GC, gastric cancer; NSAIDs, non-steroidal antiinflammatory drugs; OR: odd ratios; RR, relative ratios; SES, social economic status; H. pylori, Helicobacter pylori.

**TABLE 3:** Healthy dietary patterns and risk and survivor of ovarian cancer: Assessment of study quality.

References	Selection				Comparability		Outcome			Score
	1	2	3	4	5A	5B	6	7	8	
**Cohort**										
Sasamoto et al. (15)	*	*	*	*	*	–	*	*	*	8
Chang et al. (17)	*	*	*	*	*	–	*	*	*	8
Armidie et al. (19)	*	*	*	*	*	–	*	*	*	8
Wen et al. (20)	*	*	*	*	*	*	*	*	*	9
Xie et al. (22)	*	*	*	*	*	*	*	*	*	9
Arthur et al. (23)	*	*	*	*	*	–	*	*	*	8
Al Ramadhani et al. (33)	*	*	*	*	*	–	*	*	*	8
Arthur et al. (34)	*	*	*	*	*	*	*	*	*	9
Cao et al. (35)	*	*	*	*	*	–	*	*	*	8
Chen et al. (36)	*	*	*	*	*	*	*	*	*	9
Liu et al. (37)	*	*	*	*	*	*	*	*	*	9
Thomson et al. (38)	*	*	*	*	*	*	*	*	*	9
**Case-control**										
Kolahdooz et al. (16)	*	*	*		*	*	*	*		7
Edefonti et al. (18)	*	*	*		*	*	*	*		7
Qin et al. (21)	*	*	*		*	*	*	*		7
Chandran et al. (39)	*	*	*	*	*	–	*	*	*	8

*For case-control studies, 1 indicates cases independently validated; 2, cases are representative of population; 3, community controls; 4, controls have no history of ovarian cancer; 5A, study controls for the most important factor; 5B, study controls for additional factors, e.g., cigarette smoking body mass index, total energy intake; 6, ascertainment of exposure by secure record or blinded interview or record; 7, same method of ascertainment used for cases and controls; and 8, the same for cases and controls. For cohort studies, 1 indicates exposed cohort truly representative; 2, non-exposed cohort drawn from the same community; 3, ascertainment of exposure by secure record (e.g., surgical records) or structured interview; 4, outcome of interest was not present at start of study; 5A, study controls for the most important factor; 5B, study controls for additional factor(s); 6, assessment of outcome is based on independent blind assessment or record linkage; 7, follow-up long enough (≥ 5 years) for outcomes to occur; and 8, adequacy of follow up of cohorts (all participants complete follow up or > 90% participants complete follow up).

### Healthy dietary patterns and ovarian cancer morbidity

**FIGURE 2:**
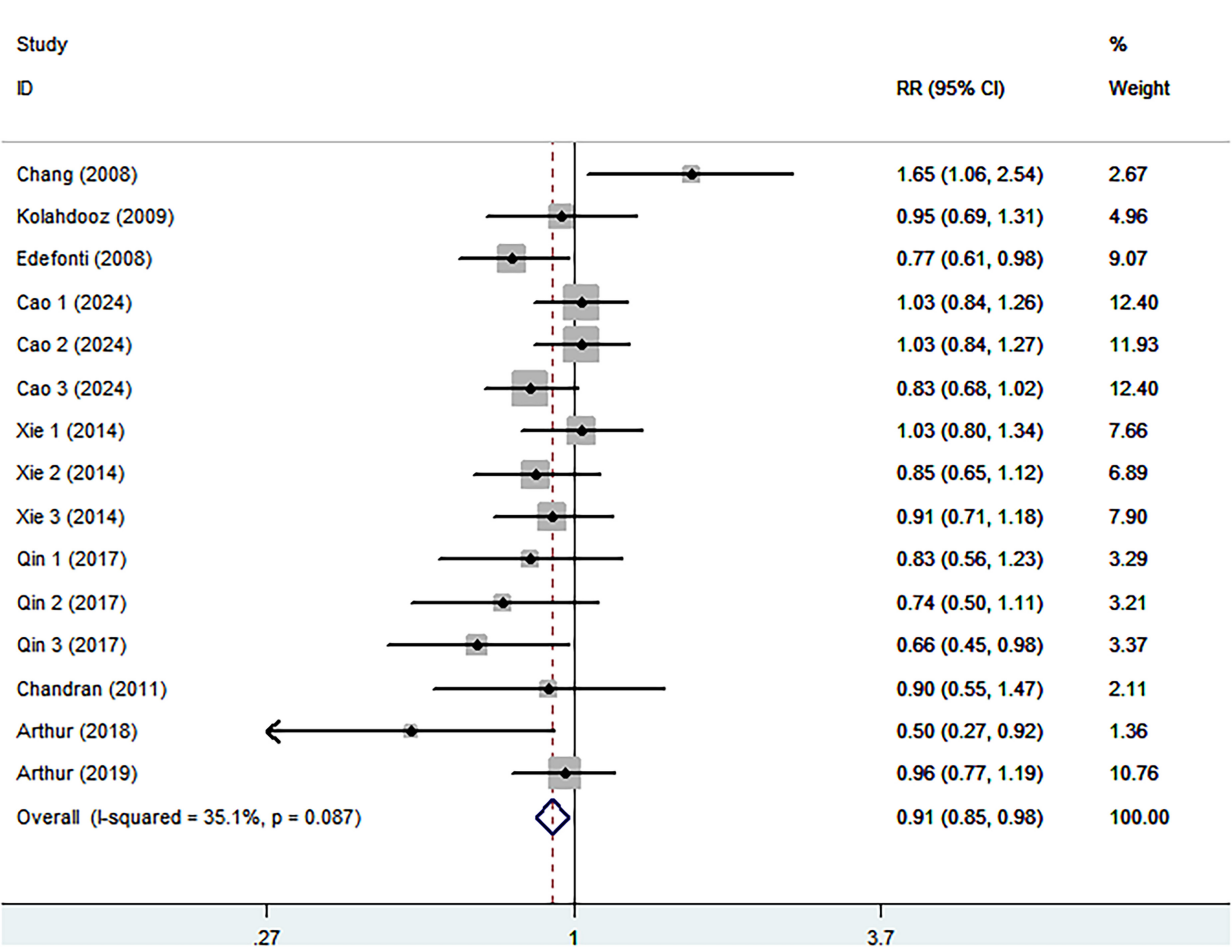
Forest plot for relative risks (RRs) of the highest compared with the lowest categories of intake of healthy dietary pattern and ovarian cancer.

Eight studies (four case-control and four cohort studies), involving 449,297 participants and 5,452 ovarian cancer cases, were included in this meta-analysis. Combining 15 effect sizes from eight studies, Figure 2 indicated the evidence of a reduced risk of ovarian cancer in the highest compared with the lowest categories of healthy dietary patterns (RR = 0.91; 95% CI:0.0.85–0.98, *P* = 0.013). The low heterogeneity was observed among the included studies (I^2^ = 35.1%, *P* = 0.087), prompting us to use a fixed-effects model.

### Healthy dietary patterns and ovarian cancer survival

**FIGURE 3:**
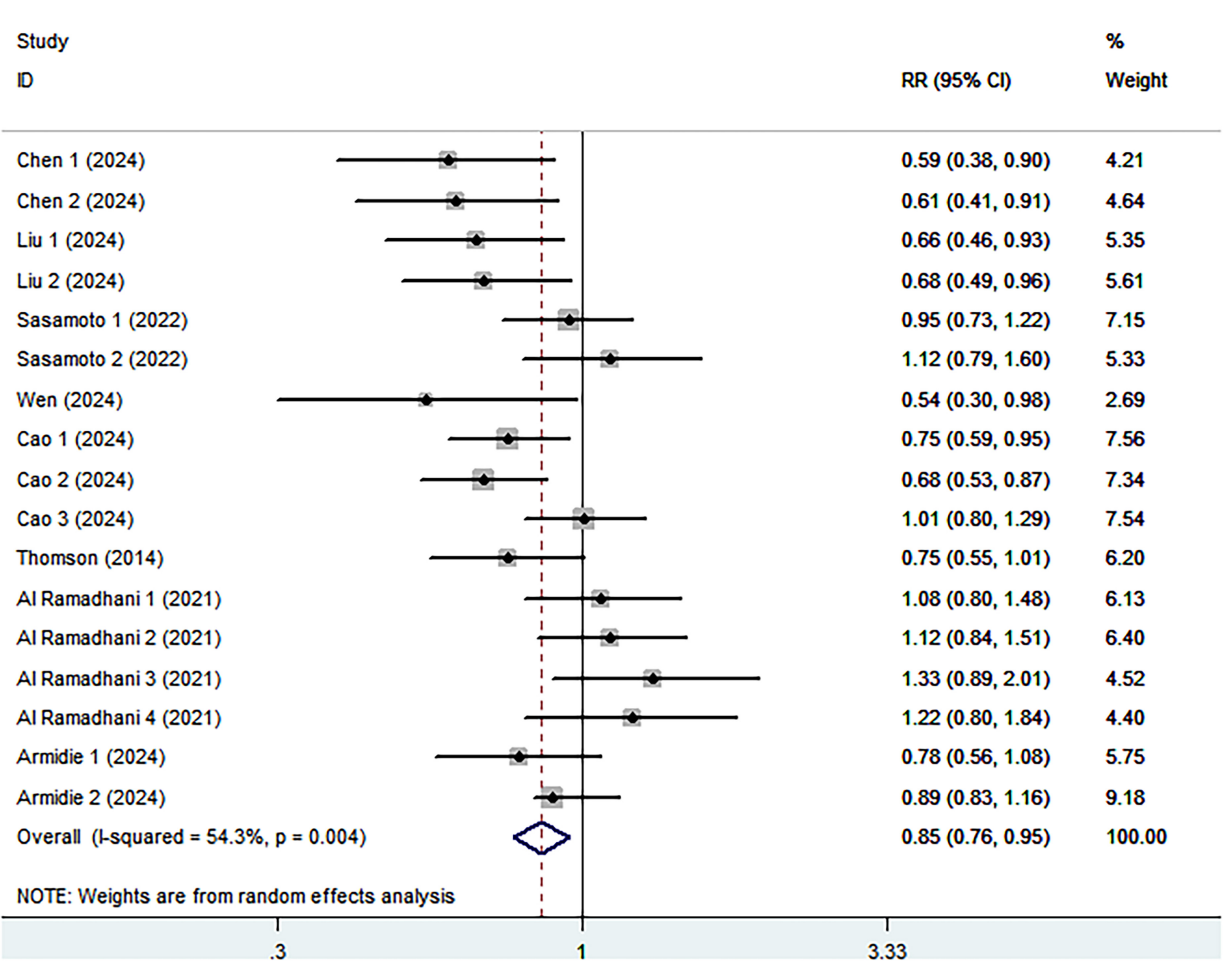
Forest plot for relative risks (RRs) of the highest compared with the lowest categories of intake of healthy dietary pattern and ovarian cancer survival.

**FIGURE 4:**
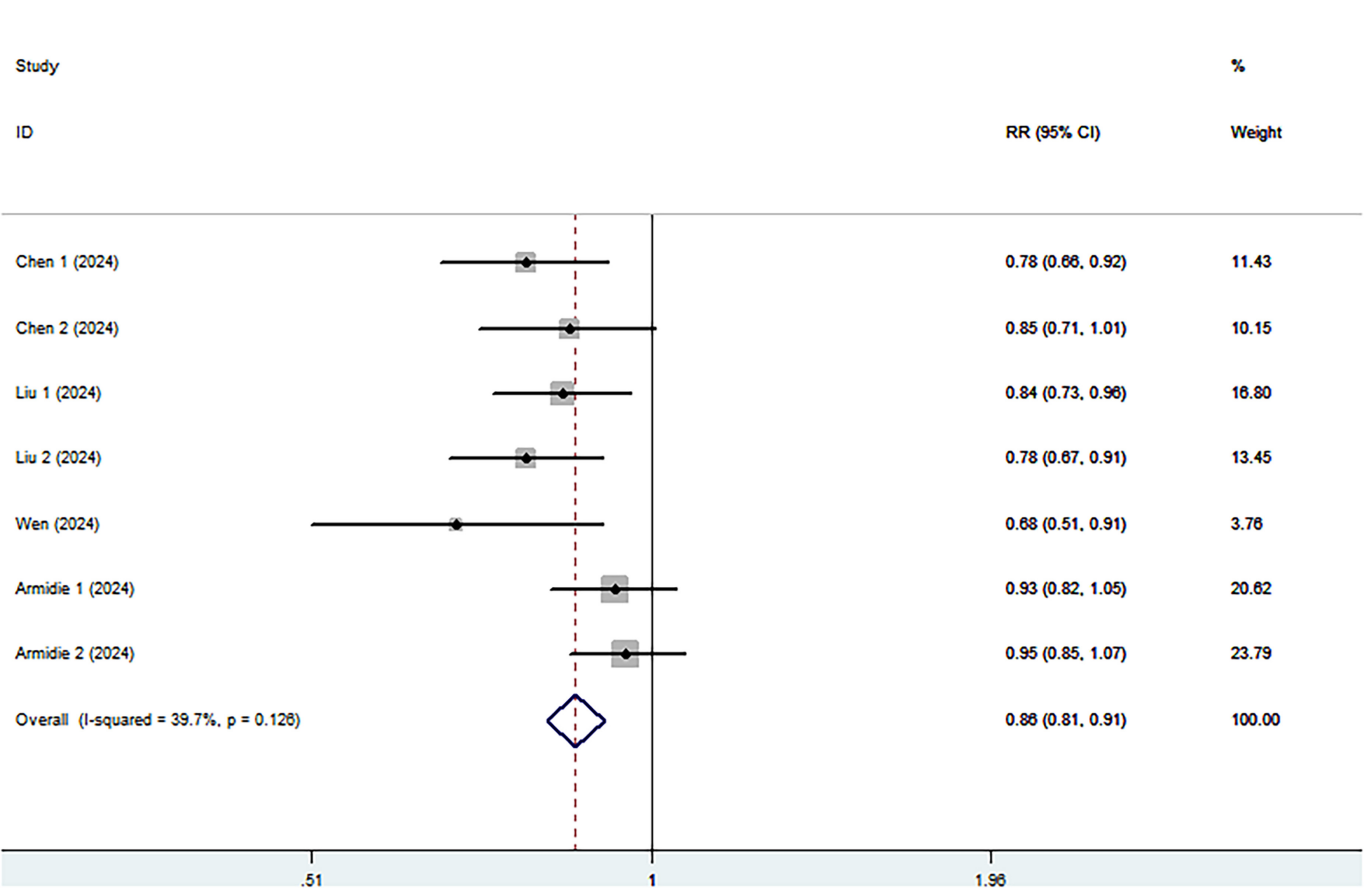
Forest plot of the association between each 1 SD increment in healthy dietary score and survival of ovarian cancer.

Eight cohort studies including 3,028 ovarian cancer deaths were included in the highest compared with lowest category meta-analysis. The association between the highest compared with the lowest categories of healthy dietary patterns with ovarian cancer survival is shown in Figure 3. Combining seventeen effect sizes from eight studies, we found that healthy dietary patterns was associated with improved ovarian cancer survival (RR = 0.85; 95% CI:0.76–0.95, *P* = 0.004), with significant heterogeneity (I^2^ = 54.3%, *P* = 0.004). As such, the effect size was assessed using a ransom-effects model. Meanwhile, Figure 4 showed that every 1 SD increment in healthy dietary score was related to a 14% reduced risk of ovarian cancer mortality (RR = 0.86, 95% CI:0.81–0.91; I^2^ = 39.7%; *P* = 0.126).

### Subgroup analyses

Given the significant heterogeneity for the association between healthy dietary patterns and ovarian cancer survival, we carried out subgroup analyses to better investigate the possible reasons (Table 4). In this study, subgroup analyses were stratified basing on study region (Asian and Western countries), methods used to determine healthy dietary patterns (*a priori* and *a posteriori*), follow-up time (< 10 and ≥ 10 years), mean age (< 50 and ≥ 50 years), and sample size (< 5,000 and ≥ 5,000). The results of subgroup analyses demonstrated an inverse association between healthy dietary patterns and survivor of ovarian cancer in the studies with mean age < 50 (RR = 0.60, 95% CI: 0.45–0.80, *P* = 0.001) and in Asian countries (RR = 0.63, 95% CI: 0.53–0.75, *P* < 0.001), with no evidence of heterogeneity (I^2^ = 0.0%).

**TABLE 4:** Subgroup analyses for the association between healthy dietary patterns and ovarian cancer survivor.

Study characteristic	Category	No. of studies	RR (95% CI)	*P-*values	Heterogeneity		
					*P-*values for within groups	I^2^ (%)	*P-*values for between groups
Study region	Western countries	5	0.93 (0.83–1.03)	0.174	0.035	47.2	< 0.001
	Asian countries	3	0.63 (0.53–0.75)	< 0.001	0.959	0.0	–
Methods used to determine healthy dietary patterns	Priori	7	0.88 (0.77–0.96)	0.008	0.005	54.0	0.123
	Posteriori	1	0.54 (0.30–0.98)	0.041	–	–	–
Follow-up time	≥ 10 years	3	0.85 (0.73–0.99)	0.043	0.078	49.5	0.772
	< 10 years	5	0.84 (0.72–0.99)	0.041	0.005	60.0	–
Sample size	≥ 5,000	2	0.79 (0.66–0.95)	0.010	0.124	47.9	0.155
	< 5,000	6	0.87 (0.76–1.00)	0.057	0.007	55.9	–
Mean age	≥ 50 years	7	0.88 (0.79–0.98)	0.024	0.011	51.6	0.014
	< 50 years	1	0.60 (0.45–0.80)	0.001	0.911	0.0	

CI, confidence interval; RR, relative risk; Y: year.

### Publication bias

No evidence of publication bias was found by visual inspection of the funnel plot (Supplementary Figures 1, 2). Moreover, Begg’s and Egger’s tests for publication bias were not statistically significant (highest compared with lowest intake: morbidity Begg’s test: *P* = 0.181, Egger’s test: *P* = 0.347; survival Begg’s test: *P* = 0.711, Egger’s test: *P* = 0.575), showing that the results were relatively stable.

### Sensitivity analysis

Based on the results of sensitivity analyses (Supplementary Figures 3, 4), we observed that no particular study had the significant effect on the associations between healthy dietary patterns and risk and survival of ovarian cancer

## Discussion

To our knowledge, this is the first systematic review and meta-analysis to comprehensively ascertain the relationship between healthy dietary patterns and ovarian cancer risk and survival. In this study, we observed that healthy dietary patterns was associated with a reduced risk and improved survival of ovarian cancer. However, significant heterogeneity was observed for the association between healthy dietary patterns and ovarian cancer survival, these results should be interpreted with caution. Collectively, our findings extend epidemiological evidence for the associations between healthy dietary patterns and improved survival of ovarian cancer, and emphasize the benefit of adhering to the healthy dietary patterns for the prevention of ovarian cancer.

Although the incidence of ovarian cancer is relatively low, it is still one of the most common gynecologic malignancies, and has the highest mortality rate in women worldwide (2). Considering the tremendous burden on public health, it is crucial to explore potentially modifiable risk factors, such as dietary factors, for the prevention of ovarian cancer. As far as we aware, diet has been recognized as an important risk factor for ovarian cancer (6). It is important to note that previous studies have mainly focused on the effects of intake of individual nutrients, foods or food groups on ovarian cancer, yielding inconclusive results (7–9). Furthermore, less is known about the associations between healthy dietary patterns and survival of ovarian cancer. Until 2014, Thomson and colleagues published the first prospective cohort study on the association between diet quality and ovarian cancer survival in the Women’s Health Initiative Observational Study (38). Since then, numerous epidemiological studies have been published to report the association between diet and survival of ovarian cancer (15, 19, 20, 33, 35, 36, 37), but the results from these published studies are entirely inconsistent. For example, Liu et al., in a prospective cohort study, found that high pre- and post-diagnosis diet quality based on the Healthy Eating Index-2020 (HEI-2020) was associated with improved OC survival (37). On the contrary, a recent cohort study used data from the African American Cancer Epidemiology Study showed that dietary quality as evaluated by HEI-2020 was not associated with ovarian cancer survival (19). In the present study, our findings revealed that adherence to the healthy dietary patterns, including HEI-2020 was significantly associated with improved survival of ovarian cancer. The discrepant results from previous studies may be attributed the following several reasons. First, there were significant differences in eating habits and lifestyle among different countries. In our analyses, fourteen of the included studies were conducted in Western countries (15–23, 33–35, 38, 39), and the remaining two studies in China (36, 37). It is well-known that there are obvious differences between Eastern and Western diets. Second, discrepancies of follow-up time in different cohort studies might explain part of these discrepant results. For instance, in Cao et al.’ study, s. the median follow-up time was 20.5 years (35), while Liu et al. included 549 ovarian cancer cases with a median follow-up of 44.9 months, representing 206 total deaths (37). Third, discrepant findings across studies might be due to insufficient sample sizes. Thomson et al.’ study had a larger sample sizes (161,808 participants), which could provide greater statistical power to identify the association between diet and ovarian cancer (38). By contrast, Armidie et al.’ study only included 483 participants (19). Fourth, inconsistent findings might also be primarily attributed to different adjustments for potential confounders in all included studies. Taken together, differences in eating habits, lifestyles, follow-up time and adjustment for confounding factors might contribute to the inconsistent results in published studies.

Even though existing evidence on the associations between healthy dietary patterns and risk and survival of ovarian cancer is inconsistent, several underlying mechanisms were possibly related to the observed favorable associations. First, vegetables and fruits are two common components of healthy dietary patterns. A recent systematic review and dose-response meta-analysis by Li et al., showed that high consumption of cruciferous vegetables were associated with a lower risk of ovarian cancer (40). In addition, as we know, vegetables and fruits are good source of dietary fiber. Earlier studies have documented an inverse association between dietary fiber intake and ovarian cancer risk (41). Furthermore, high consumption of dietary fiber has been suggested to be inversely associated with risk of obesity, an important risk factor for ovarian cancer (42). Second, healthy dietary patterns were often characterized to have a low consumption of animal fat and meat, in particular processed meat. A previous meta-analysis showed an increased risk of ovarian cancer for the highest vs. lowest intake of total fat, animal fat and saturated fat (43). Moreover, processed meat is known to contain high levels of salt, nitrites or nitrosamine compounds, which are all thought to be carcinogenic (44). Third, the beneficial effects of healthy dietary patterns on ovarian cancer may be related to cooking methods. Increasing evidence suggests that cooking meat, especially at high temperature, e.g., pan-frying or grilling can produce large amounts of heterocyclic aromatic amines, or polycyclic aromatic hydrocarbons, which are thought to be carcinogenic (45). Fourth, vegetables and fruits are also good sources of antioxidants, such as vitamin C and carotenoids, which may neutralize reactive oxygen species and prevent free radical damage in carcinogenic process (46). Additionally, these foods also provide a good source of folate. Previous studies have shown that folate plays a key role in the repair, synthesis and methylation of DNA, thereby preventing carcinogenesis (47). As already discussed above, these mechanisms could together explain the favorable associations observed between healthy dietary patterns and ovarian cancer risk and survival.

Notably, significant heterogeneity (I^2^ = 54.3%, *P*_*heterogeneity*_ = 0.004) was found in the association between healthy dietary patterns and ovarian cancer survival. Although heterogeneity is common in published meta-analyses (11, 48), it is critical to characterize potential sources of statistical heterogeneity. Therefore, we performed subgroup analyses with respect to study region (Asian and Western countries), methods used to determine healthy dietary patterns (*a priori* and *a posteriori*), follow-up time (< 10 and ≥ 10 years), mean age (< 50 and ≥ 50 years), and sample size (< 5,000 and ≥ 5,000). When we analyzed by study region and mean age separately, heterogeneity decreased from 54.3% to 0.0%. Thus, the subgroup analyses revealed that study region and mean age were the potential sources of significant heterogeneity. On the one hand, there were significant differences in Eastern and Western countries. In our study, the vast majority of the included studies were conducted in Western countries (15–23, 33–35, 38, 39) and the remaining two studies were conducted in Eastern countries (36, 37). On the other hand, younger participants were more likely to choose a variety of foods (49). Thus, they would not only choose some healthy foods, such as vegetables, fruits, and whole grains etc., but also choose high-energy foods, such as sugar-sweetened drinks, crisps, cookies and cakes. Along with the above-mentioned, four of all included studies were case-control studies, and recall and selection bias might at least partially explain the significant heterogeneity.

### Strengths and limitations

This study has several advantages. First, this is the first systematic review and meta-analysis to comprehensively assess the associations between healthy dietary patterns and the risk and survival of ovarian cancer. Our findings add epidemiological evidence for the association between healthy dietary patterns and improved survival of ovarian cancer, and highlight the importance of adherence to the healthy dietary patterns for the prevention of ovarian cancer. Second, ovarian cancer cases were diagnosed through view of cancer registry or medical records or pathological records, avoiding misdiagnosis bias. Third, the inclusion of a large number of participants and ovarian cancer cases gives robustness to the results. Fourth, subgroup and sensitivity analyses were carried out to explore the possible sources of heterogeneity, which increase confidence in the findings. Fifth, no signs of publication bias were evident in the funnel plot, and Begg’s and Egger’s tests for publication bias were non-significant. Finally, we performed a rigorous literature screening based on inclusion and exclusion criteria. Despite the strengths, some limitations should also be taken into account when interpreting our meta-analysis findings. First, all included studies are observational design, so causality cannot be established. Thus, further studies especially with prospective design are needed to provide evidence for the causal relationship. Second, all eligible studies used FFQs to collect dietary information, which are prone recall bias and to under- or over-estimation of healthy foods intake. Third, there was significant heterogeneity for the association between healthy dietary patterns and ovarian cancer survival. Although subgroup analyses showed that differences in study region and mean age can partially explain the observed heterogeneity, the results still need cautious interpretation. Fourth, although majority of included studies have adjusted for potential confounding factors, residual and unmeasured confounding cannot be ignored in observational studies. Fifth, we could not perform the dose-response analysis, due to the limited data reported in the included studies. Finally, the current study had a geographical restriction, because most of the included studies were performed in the Western population, e.g., United States, where eating habits and food culture were significantly different from those in Asian population. Such geographical differences limited the generalizability of our study findings to other populations. Accordingly, more studies, particularly in different populations, are warranted to validate the correlation between healthy dietary patterns and ovarian cancer risk and survival.

## Conclusion

In summary, this study showed that adherence to the healthy dietary patterns was associated with a reduced risk and improved survival of ovarian cancer. These results are agreement with previous findings and underscore the importance of adherence to the healthy dietary patterns for the prevention of ovarian cancer. Besides, our findings also support public health recommendations that encourage the adoption of healthy dietary patterns. Nevertheless, considering all the above limitations, additional large prospective studies, particularly from Asian and African regions, are warranted to confirm these associations in the future.

## Supplementary material

Supplementary Figure 1 Funnel plot for the association between healthy dietary patterns and ovarian cancer morbidity.

Supplementary Figure 2 Funnel plot for the association between healthy dietary patterns and ovarian cancer survival.

Supplementary Figure 3 Sensitivity analysis for the association between healthy dietary patterns and ovarian cancer morbidity.

Supplementary Figure 4 Sensitivity analysis for the association between healthy dietary patterns and ovarian cancer survival.
