# Supplementary material for: Healthy dietary patterns and ovarian cancer risk and survival: a systematic review and meta-analysis
Source: Front Nutr. 2025 Oct 13;12:1681162. doi: 10.3389/fnut.2025.1681162 (PMC12554572; doi:10.3389/fnut.2025.1681162)
Supplement: Supplementary file 1 [file Table_1.doc]

**Search terms in PubMed, Scopus, Embase and Web of Science**
(“diet”[all fields] OR “dietary score” [all fields] OR “diet indices”[all fields] OR “dietary quality”[all fields] OR “dietary index”[all fields] OR “dietary pattern”[all fields] OR “eating pattern”[all fields] OR “food pattern”[all fields]) AND (“ovarian neoplasm”[all fields] OR “ovarian cancer”[all fields] OR “ovarian carcinoma”[all fields] OR “ovarian tumor”[all fields] OR “ovarian mass”[all fields] OR ““ovary neoplasm”[all fields] OR “ovary cancer”[all fields] OR “ovary carcinoma”[all fields])

**Search terms in China National Knowledge Infrastructure(CNKI)**

(膳食模式 OR 饮食) and 卵巢癌

**Search terms in other sources**

The reference lists of retrieved studies, published reviews and meta-analyses were also manually examined to identify additional relevant studies.
