# Supplementary figures and images for: Healthy dietary patterns and ovarian cancer risk and survival: a systematic review and meta-analysis
Source: Front Nutr. 2025 Oct 13;12:1681162. doi: 10.3389/fnut.2025.1681162 (PMC12554572; doi:10.3389/fnut.2025.1681162)

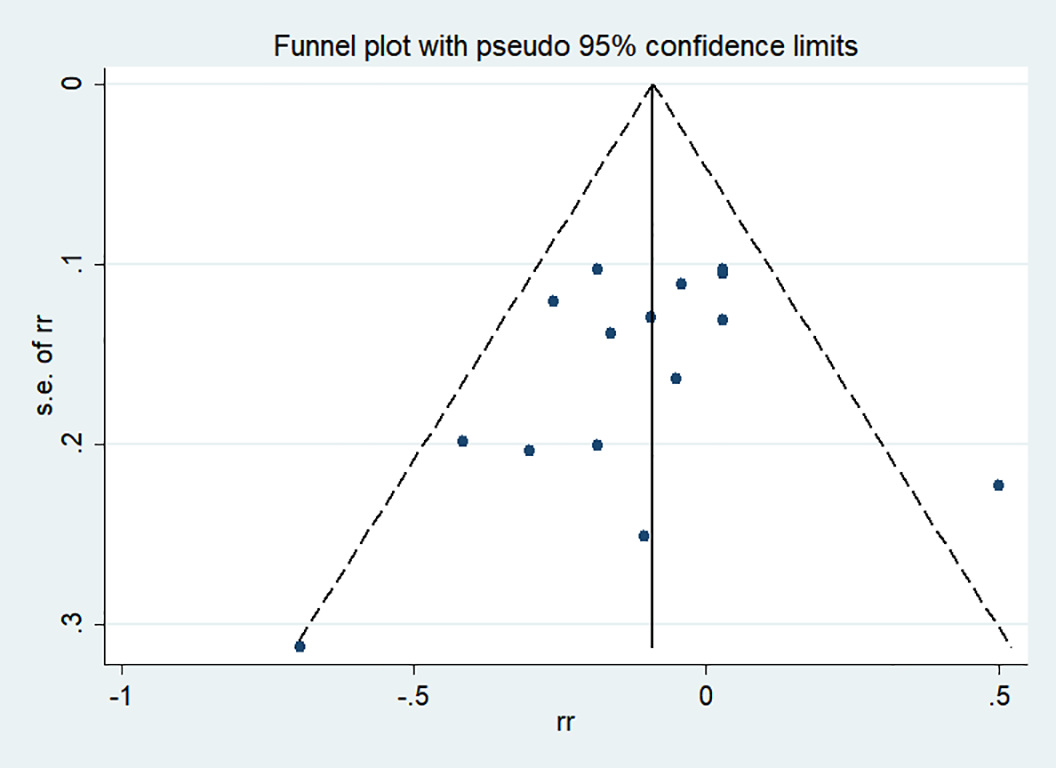

Supplement: Supplementary Figure 1 — Funnel plot for the association between healthy dietary patterns and ovarian cancer morbidity. [file Image_1.tif]

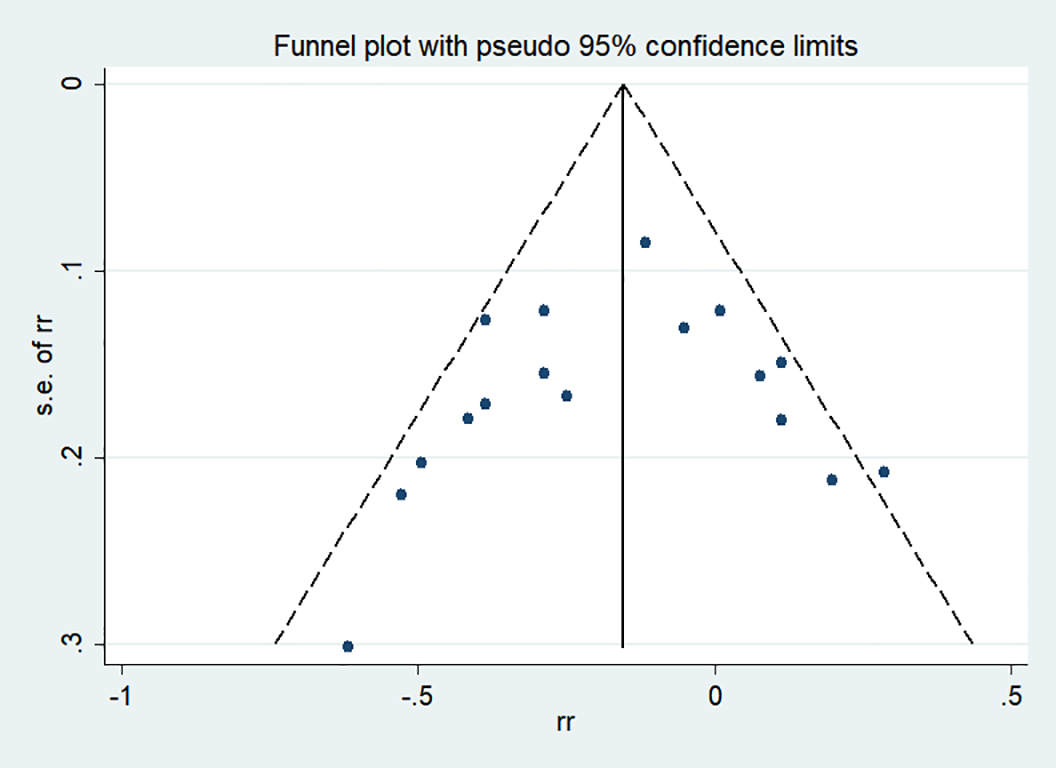

Supplement: Supplementary Figure 2 — Funnel plot for the association between healthy dietary patterns and ovarian cancer survival. [file Image_2.tif]

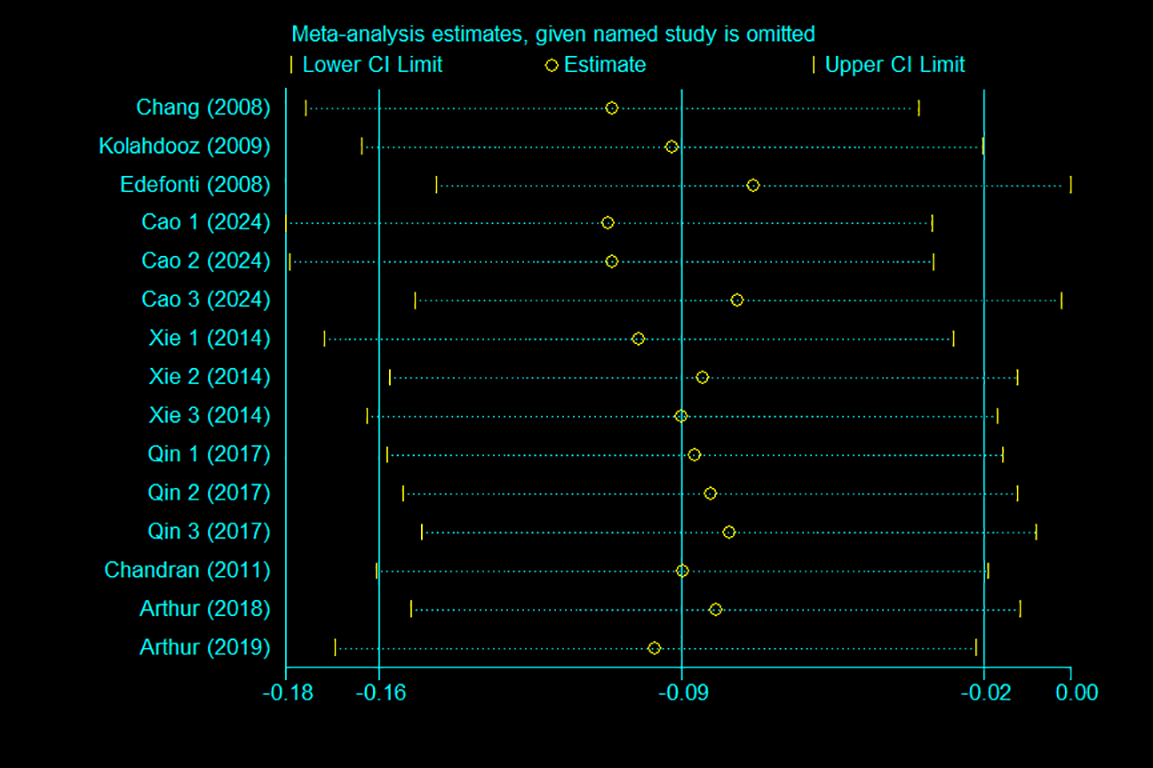

Supplement: Supplementary Figure 3 — Sensitivity analysis for the association between healthy dietary patterns and ovarian cancer morbidity. [file Image_3.tif]

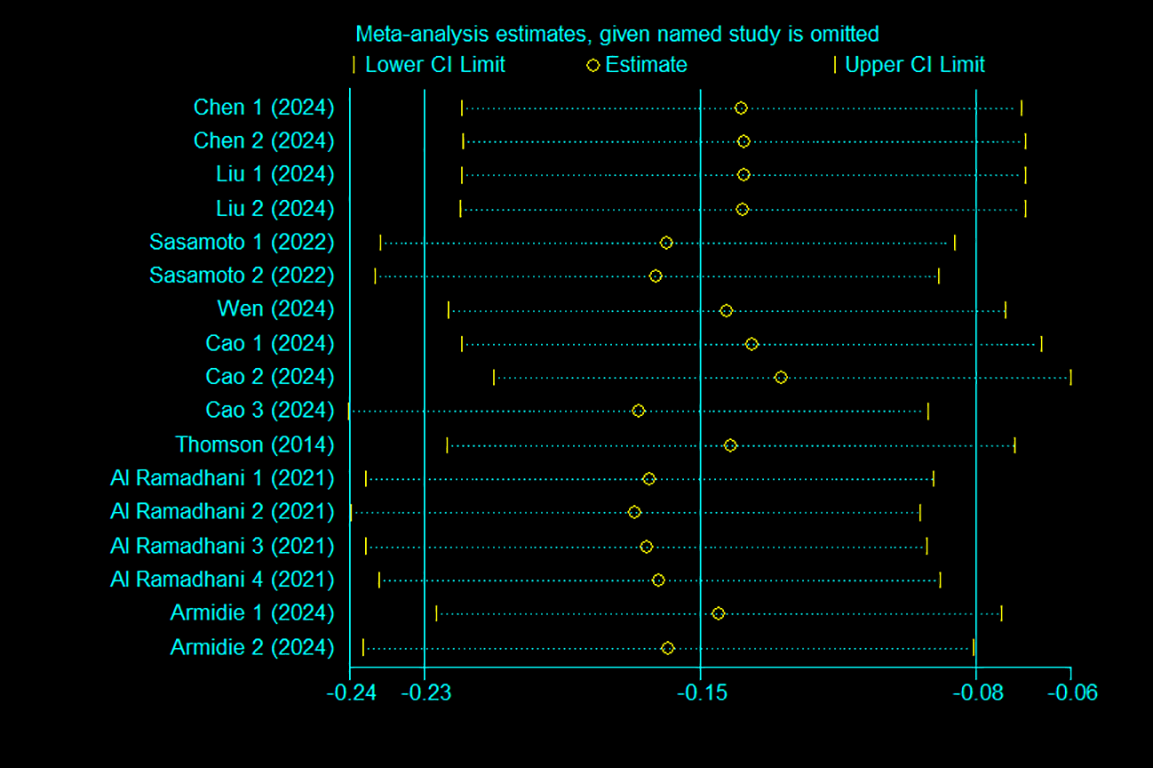

Supplement: Supplementary Figure 4 — Sensitivity analysis for the association between healthy dietary patterns and ovarian cancer survival. [file Image_4.tif]
